# Beyond the cities: socioemotional mediators of the link between childhood maltreatment and academic outcomes in rural Chinese adolescents

**DOI:** 10.3389/fpsyg.2026.1786513

**Published:** 2026-04-10

**Authors:** Xiaohui Sun, Yu Xue, Zhiyu Chen, Hongzhu Sun

**Affiliations:** 1 University of Dundee, Dundee, United Kingdom; 2 Washington State University, Pullman, WA, United States; 3 University of Emergency Management, Sanhe, China

**Keywords:** academic performance, childhood maltreatment, Chinese rural adolescents, empathy, prosocial behavior, socioemotional development

## Abstract

Childhood maltreatment has been evidenced compromising academic outcomes, yet mechanisms remain unclear, particularly in rural populations facing educational inequalities. This study examined whether socioemotional capacities of empathy and prosocial behavior mediate maltreatment’s association with academic performance among 547 rural Chinese adolescents (mean age = 14.475; 51.2% male) based on cross-sectional data. Participants completed measures of childhood maltreatment, empathy, prosocial behavior, and academic performance. Structural equation modeling tested a chain mediation model. Results revealed that maltreatment directly associated with poorer academic performance. Prosocial behavior independently mediated this relationship, while empathy alone did not show significant mediation. However, a sequential pathway from empathy through prosocial behavior to academic performance was significant. Findings suggested that maltreatment disrupted academic achievement partially through impaired socioemotional development, with behavioral manifestations (prosocial behavior) more proximally linked to academic outcomes than internal emotional capacities (empathy) alone. This study suggested that interventions for prosocial skills and translation from internal empathetic concern to external prosocial actions, as well as maltreatment prevention were potentially helpful to support educational outcomes in vulnerable rural adolescents. These findings extended Western-based theories to the Chinese rural context and underscored socioemotional pathways as intervention targets for mitigating maltreatment’s educational consequences.

## Introduction

Childhood maltreatment, encompassing physical, emotional, and sexual abuse, as well as physical and emotional neglect, represents harmful caregiver behaviors threatening child development (World Health Organization, 2024). In China, 20–47% of children experience various types of maltreatment, with documented harmful neurobiological and academic consequences (Wang et al., 2020). While research links maltreatment to academic difficulties via executive dysfunction and emotional dysregulation (Romano et al., 2015), the socioemotional mechanisms underlying this relationship remain less understood.

Academic performance is a cornerstone of adolescent development and future opportunities, predicting higher education, employment, income, and social mobility (OECD, 2010; Steindórsdóttir et al., 2024). Poorer performance increases the risks of school dropout, behavioral maladjustment, unemployment, as well as other socioeconomic disadvantages (Rumberger, 2011; Lansford et al., 2016; Lankester et al., 2024). In China, where *Gaokao* (national college entrance examination) largely defines social mobility (Li, 2012; Zhao, 2013), rural students face acute challenges such as limited educational resources, underqualified teachers, and reduced parental support due to labor migration brought by deficient local employment opportunities (Li et al., 2017; Hung, 2019).

Beyond these structural disparities, childhood maltreatment represents a particularly critical yet underexplored factor. With a 2.45:1 urban–rural income disparity (National Bureau of Statistics of China, 2025) and the Gaokao-dominated education system in China, understanding how maltreatment undermines academic performance for rural areas becomes critical. Unlike poverty or lower parental education, which primarily limits material resources, maltreatment directly undermines socioemotional development through psychological pathways. This distinction is especially salient in rural China, where parental migration has increased neglect risk and limited community safety nets elevate adolescents’ vulnerability to maltreatment (Chen et al., 2020). Despite its importance, little empirical work examines how childhood maltreatment influences rural Chinese adolescents’ educational outcomes.

Two socioemotional constructs, empathy and prosocial behavior, present potential explanatory pathways. Adolescence is marked by heightened socioemotional sensitivity (Casey et al., 2011). At the emotional level, empathy develops through secure attachment relationships that maltreatment can disrupt (Bowlby, 1969; Berzenski and Yates, 2022). During adolescence, when abstract thinking emerges and peer relationship intensifies, empathic deficits become particularly consequential for academic engagement through impaired teacher-student bonds and collaborative learning (Marilyn and Marr, 2022; Wang et al., 2022). At the behavioral level, prosocial behavior, as a more observable socioemotional competence, enhances peer acceptance and teacher support in collaborative learning environments (Wentzel, 2014). The empathy-altruism hypothesis suggests these mechanisms as sequential, with emotional understanding preceding helping behaviors (Batson, 1987). This relationship tends to be amplified during adolescence, as individuals’ moral reasoning starts to shift from external to internal sources (Eisenberg, 2000).

Although maladaptive consequences of childhood maltreatment are well-documented (May et al., 2022), the socioemotional processes explaining educational disadvantages remain underexplored, compared to cognitive or resilience-based explanations (Ferrara and Panlilio, 2020; Gresham and Karatekin, 2023; Erduran Tekin, 2024). In China, where Confucian philosophy emphasizes interpersonal relationships and social harmony (Wei et al., 2013), these factors may exert especially strong influence. However, no integrated model has tested how childhood maltreatment influences academic performance via these pathways. Whether empathy, as a more implicit factor, requires behavioral expression to impact academic outcomes also remains untested.

This study addresses these gaps by examining the potential roles of empathy and prosocial behavior as potential mediators between childhood maltreatment and academic performance amongst rural Chinese adolescents. Four strands of literature inform this model: (a) the direct association between childhood maltreatment and academic performance, (b) the association between maltreatment and empathy/prosocial behavior, (c) the contributions of empathy and prosocial behavior to academic performance, and (d) the sequential link between empathy and prosocial behavior. Together, these perspectives provide the theoretical foundation for the hypothesized mediation model.

## Literature review

### Childhood maltreatment and academic performance

A large body of international research has consistently shown a negative association between childhood maltreatment and academic outcomes, including lower grade point averages, weaker reading comprehension, reduced motivation, and higher rates of absenteeism and school dropout (Slade and Wissow, 2007; Hagborg et al., 2018; Ferrara and Panlilio, 2020). These associations have been explained through several theoretical perspectives. From an attachment theory lens (Bowlby, 1969), maltreatment disrupts secure caregiver bonds, which are essential for developing emotional regulation, trust, and persistence in learning contexts (Dvir et al., 2014; Cooke et al., 2019; Ben-Gal Dahan and Mikulincer, 2021). The toxic stress framework (Shonkoff et al., 2012) further posits that chronic exposure to abuse or neglect alters neurobiological systems compromising core academic competencies. Such exposure elevates cortisol levels, impairing hippocampal memory consolidation and prefrontal executive functions, which compromise individuals’ working memory, sustained attention, and cognitive flexibility, all critical for academic learning (Cowell et al., 2015; Kirke-Smith et al., 2014; Shonkoff et al., 2012). Simultaneously, amygdala hyperactivation redirects cognitive resources toward threat detection, thereby depleting the attentional resources available for learning (Shonkoff et al., 2012).

Studies in Western contexts demonstrate that children with maltreatment histories are more likely to experience poorer academic performance. However, much less is known about how these processes unfold in China grounded in the large population size, high prevalence of childhood maltreatment, and particular importance attached on academic performance (Bai et al., 2022; Li et al., 2022; Wang et al., 2020). Existing studies suggest that the combination of maltreatment and rural educational disadvantages may create cumulative risks for adolescents, amplifying academic disparities with urban areas (Li et al., 2024). Yet, few investigations have explicitly modeled the pathways through which maltreatment undermines educational performance in rural Chinese samples. Based on the above discussion, we propose

*H1*: Childhood maltreatment is negatively associated with academic performance.

### Mediating role of empathy

In addition to academic outcomes, maltreatment profoundly undermines socioemotional development influencing relationships with others. A growing body of evidence indicates that children with abuse/neglect exposure develop significant deficits in empathy. Specifically, they exhibit greater difficulty in identifying emotional expressions, expressing empathic concern, and engaging in perspective-taking (Zhang H. et al., 2024a; Zhang et al., 2024). Neurobiological studies suggest that early adversity alters the structure and function of brain regions involved in emotion regulation and social cognition essential for empathic capacities, such as the amygdala and prefrontal cortex (Decety, 2011). From an attachment theory perspective (Bowlby, 1969), maltreatment disrupts secure bonds with caregivers, fostering insecure or disorganized attachment styles. Children’s ability to trust and appropriately respond to others’ emotions would be limited (Bifulco et al., 2002). Similarly, trauma theory highlights emotional numbing and hypervigilance as survival strategies, which blunt empathic responsiveness and make maltreated youth less likely to show concern for others’ suffering (Bevan and Higgins, 2002).

Beyond cognitive skills, empathy has emerged as potential predictor for academic success. Adolescents with higher levels of empathy tend to establish closer and more supportive relationships with teachers and peers, which would further foster stronger school belonging and academic engagement (Dearth-Wesley et al., 2023; Tikkanen et al., 2024). Empathic students are also more able to manage interpersonal conflicts and collaborate in group learning, contributing to higher grades and persistence in school directly (Van Ryzin and Roseth, 2019). In Chinese context, where harmony and relational sensitivity are highly valued, empathy may be particularly important for teacher evaluations and peer relationship, both influencial for academic outcomes (Chan and Lam, 2023). The above discussion suggests empathy as a critical socioemotional pathway through which adolescents achieve academic success, which generates

*H2*: Empathy mediates the association between childhood maltreatment and academic performance.

### Mediating role of prosocial behavior

Maltreatment also exerts a detrimental influence on prosocial behavior. Research consistently shows that abused or neglected children are less likely to engage in prosocial acts such as sharing, helping, or cooperating with peers (Prior et al., 2021). According to social cognitive theory (Bandura, 1986), which extends earlier social learning theory (Bandura et al., 1961), children acquire behavioral repertoires not only through observational learning but also through cognitive processes such as internalized beliefs, expectations, and self-construals shaped by environments. In adverse caregiving contexts, adolescents may develop maladaptive social expectations and emotional schemas, which reduce empathic responsiveness and prosocial behavioral tendencies. More recent empirical studies also suggest that maltreated youth are more likely to experience peer rejection, which reduces opportunities for reciprocal prosocial interactions and further entrenches antisocial patterns (Dye, 2020).

As a behavioral socioemotional capability, prosocial behavior has been suggested with positive link to school success across diverse cultural settings. Students who regularly help, share, and cooperate with peers are more likely to be get involved in their social networks, receive reciprocal support, and demonstrate higher classroom participation (Jambon and Malti, 2022). Studies have shown that prosocial conduct are associated not only with enhanced academic achievement (Gerbino et al., 2018) but also reduced emotional/behavioral problems and lower risk of school dropout (Flynn et al., 2015; Memmott-Elison and Toseeb, 2023).

From the perspective of self-determination theory (Ryan and Deci, 2000), prosocial behavior contributes to the satisfaction of adolescents’ three basic psychological needs: relatedness, competence, and autonomy. Prosocial interactions learnt from childhood relationship with caregivers influence relatedness by fostering positive connections with peers and teachers in education settings. They can also enhance competence by reinforcing perceived efficacy in finishing challenging tasks. Moreover, prosocial behaviors reinforce autonomy, which is a sense of choice to actively solve both academic and social problems, as prosocial behaviors are typically self-initiated volition rather than externally imposed (Ryan and Deci, 2000). The satisfaction of these psychological needs promotes intrinsic motivation, engagement, and persistence in academic activities, thereby contributing to improved academic performance. Moreover, prosocial behavior strengthens school connectedness, a known protective factor that promotes engagement and buffers the negative impact of adverse experience (Goetschius et al., 2023). Given all the above discussion, we develop

*H3*: Prosocial behavior mediates the association between childhood maltreatment and academic performance.

### The chain mediation from empathy to prosocial behavior

Finally, a robust body of evidence demonstrates that empathy is a key antecedent of prosocial behavior. The empathy–altruism hypothesis posits that empathic concern for others generates altruistic motivation and further drives prosocial acts (Batson, 1987). Developmental studies confirm that adolescents who report higher levels of empathic concern are more likely to engage in helping, cooperation, and altruistic behavior in school settings (Pang et al., 2022; Ramey et al., 2017). Prosocial behavior derived from empathy further enhances peer trust, social support, and a sense of school belonging, which in turn contribute to academic engagement (Top et al., 2016; Oberle et al., 2023). Thus, empathy and prosocial behavior are not only individually important but also sequentially linked in shaping developmental and educational outcomes, leading to the following hypothesis:

*H4*: Empathy and prosocial behavior sequentially mediate the association between childhood maltreatment and academic performance.

### The present study

Although prior research has identified a range of mediators linking childhood maltreatment to academic performance, including resilience (Erduran Tekin, 2024), coping strategies (VanMeter et al., 2020), psychological distress (Gresham and Karatekin, 2023), metacognitive processes (Ferrara and Panlilio, 2020), and social support (Lopes and Moleiro, 2011), the role of socioemotional mechanisms has been comparatively neglected. Most existing studies have focused on how cognitive or resilience-based factors enable youth to adapt to adverse experience (VanMeter et al., 2020; Erduran Tekin, 2024), yet they often overlook the interpersonal and emotional pathways. In particular, empathy and prosocial behavior, which are central to adolescents’ social adaptation, have rarely been considered as mediators in this relationship.

Among the few studies examining these constructs, empathy and prosocial behavior are often treated separately rather than jointly modeled. This limits our understanding of whether they form a sequential process, in which deficits in empathy reduce prosocial conduct and ultimately undermine academic achievement. While the empathy–altruism hypothesis (Batson, 1991) and self-determination theory (Ryan and Deci, 2000) both suggest such a pathway, empirical tests of this chain mediation model remain scarce (Yu et al., 2020). Moreover, the bulk of prior work has been conducted in Western contexts. To date, few studies have systematically tested an integrated socioemotional mediation model specifically amongst rural Chinese adolescents, a population disproportionately exposed to maltreatment (Wang et al., 2020; Li and Li, 2023). Addressing this gap is both theoretically significant because it extends socioemotional theories into the domain of academic outcomes and practically urgent for improving educational equity for vulnerable populations.

**Figure 1:**
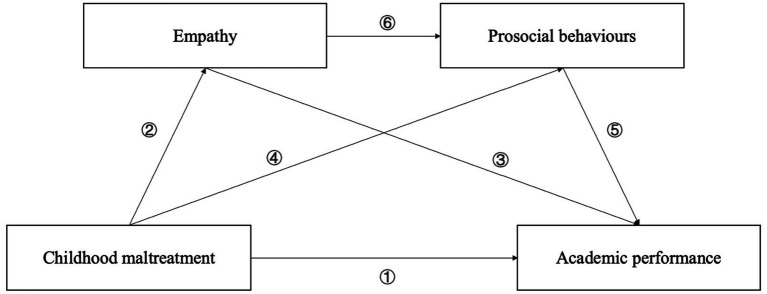
Theoretical framework. ①: Toxic stress framework ②: Attachment theory ③: Inter-personal contact theory ④: Social learning theory ⑤: Self-determination theory ⑥: Empathy-altruism hypothesis.

**Figure 2:**
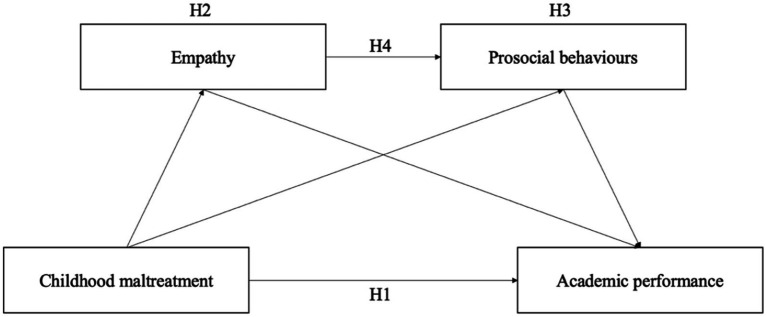
Hypothesized chain mediation model.

Figure 1 demonstrates the theoretical framework of this study, which integrates multiple complementary theories to explain how childhood maltreatment undermines academic performance through the socioemotional mechanisms of empathy and prosocial behavior. The attachment theory (Bowlby, 1969) and toxic stress framework (Shonkoff et al., 2012) explain how maltreatment disrupts emotional security and neurobiological systems essential for empathy development. The empathy–altruism hypothesis (Batson, 1991) further explains how empathy serves as a precursor to prosocial behavior by generating altruistic motivation. Social learning theory (Bandura et al., 1961) suggests that adverse caregiving environments limit opportunities to acquire adaptive interpersonal behaviors, which further reduce prosocial conduct. Finally, self-determination theory (Ryan and Deci, 2000) and interpersonal contact theory (Van Ryzin and Roseth, 2019) explain how prosocial behavior enhances academic performance by strengthening social connectedness, intrinsic motivation, and supportive relationships in educational contexts. Together, these theoretical perspectives form an integrated framework in which childhood maltreatment disrupts emotional and behavioral processes critical for academic success. Specifically, we advance the following four hypotheses (Figure 2):

*H1:* Childhood maltreatment is negatively associated with academic performance.

*H2*: Empathy mediates the association between childhood maltreatment and academic performance.

*H3*: Prosocial behavior mediates the association between childhood maltreatment and academic performance.

*H4*: Empathy and prosocial behavior sequentially mediate the association between childhood maltreatment and academic performance.

## Methods

### Participants

Participants were recruited via stratified sampling from four rural middle schools in Shandong Province, China. Each area represented a city ranked 4th, 8th, 12th, and 16th in per capita GDP among the 16 administrative regions of the province (Shandong Provincial Bureau of Statistics, 2025). A total of 605 adolescents completed a paper-and-pencil questionnaire in their classrooms under the researchers’ supervision. After data screening, which involved removing incomplete responses and cases failing attention-check items, 547 participants were retained for data analysis, yielding a valid response rate of 90.413%. Participants’ age ranged from 12 to 17 years (*M* = 14.475, *SD* = 1.058), including 280 males and 267 females. The reported demographics can be seen in Table 1.

**Table 1:** Participant demographics.

Demographic	Category	Number
Gender	Male	280
	Female	267
Parent marital condition	Divorced	24
	Not divorced	521
Sibling condition	Only child at home	36
	Had sibling(s)	488
Father’s highest education level	Primary school	47
	Middle school	405
	Vocational training	3
	High school	65
	College	12
Mother’s highest education level	Primary school	112
	Middle school	366
	Vocational training	3
	High school	37
	College	10

This study received ethics approval from the School of Marxism, University of Emergency Management. Written informed consent was obtained from both parents and students prior to participation, which was strictly voluntary. During classroom administration, trained school counselors and teachers were present to provide immediate support if any student experienced discomfort. Confidentiality was assured and students were informed that they could withdraw at any time without consequences.

### Measures

Participants completed the questionnaires in a fixed standardized order with classroom administration to ensure consistent administration across participants. Specifically, the measures were administered in the sequence of empathy, prosocial behavior, academic performance, and childhood maltreatment. This ordering is consistent with recommended practices for minimizing method bias when administering sensitive psychological measures (Podsakoff et al., 2003). Administering childhood maltreatment items after other measures helps to reduce the risk of negative priming effect, which may otherwise influence responses to subsequent constructs (Podsakoff et al., 2003).

All four primary constructs were modeled as latent variables in the SEM. Childhood maltreatment, empathy, and prosocial behavior were indicated by their respective subscale scores, whereas academic performance was indicated by standardized exam scores and self-reported academic functioning. These indicators were used to define their respective latent constructs in the SEM analysis.

### Childhood maltreatment

Childhood maltreatment was measured using the 28-item Childhood Trauma Questionnaire–Short Form (CTQ-SF; Bernstein et al., 1994). The CTQ-SF assesses five domains: emotional abuse, physical abuse, sexual abuse, emotional neglect, and physical neglect. Items were rated on a five-point Likert scale (1 = “never true,” 5 = “very often true,” e.g., “People in my family called me things like ‘stupid’, ‘lazy’, or ‘ugly’”). This questionnaire has demonstrated validity and reliability across countries and age groups, including Chinese adolescents (Infurna et al., 2024; Xie et al., 2024). In this study, the scale showed good internal consistency (Cronbach’s *α* = 0.881).

### Empathy

Empathy was assessed using the 28-item Interpersonal Reactivity Index (IRI; Davis, 1980), including four subscales: fantasy, perspective-taking, personal distress, and empathic concern. Items were rated on a five-point Likert scale (1 = “does not describe me well,” 5 = “describes me very well,” e.g., “Before criticizing somebody, I try to imagine how I would feel if I were in their place”). The IRI has demonstrated high reliability among diverse adolescent populations, including Chinese youth (Trentini et al., 2022; Pan et al., 2022). In this study, Cronbach’s *α* = 0.743.

### Prosocial behavior

Prosocial behavior was measured using the revised 26-item Prosocial Tendencies Measure (PTM; Carlo and Randall, 2002; Chinese adaptation by Yu et al., 2007). The PTM evaluates six domains: emotional, compliant, altruistic, anonymous, public, and dire prosocial behaviors. Items were rated on a five-point Likert scale (1 = “never,” 5 = “very often,” e.g., “It is most fulfilling to me when I can comfort someone who is very distressed”). The PTM has shown strong reliability across international and Chinese adolescent samples (Capella et al., 2023; Yao and Li, 2024). In this study, Cronbach’s *α* = 0.953.

### Academic performance

Academic performance was modeled as a latent construct with four indicators: standardized exam scores and three self-reported academic domains. The most recent exam scores were converted into a five-point scale based on the percentage of the total possible score (e.g., a score of 90 out of 100 corresponds to 90%, which translates to 4.5 on the 5-point scale). In addition, three previously validated questionnaires were used to assess academic competence (Alsaker, 1989), academic engagement (Benson et al., 1998), and school satisfaction (Cheung and Cheung, 2005), rated on five-point Likert scales (1 = “strongly disagree,” 5 = “strongly agree”). In the SEM, standardized exam scores and the three self-reported indexes were specified as four independent indicators of the latent academic performance construct. This modeling approach allowed the integration of both objective academic achievement and subjective academic functioning while accounting for measurement error. In this study, Cronbach’s α = 0.814.

### Data analysis

Following recommended practices, cases with excessive missing data (≥ 10% of questionnaire items) were excluded to ensure data quality and reliability (Enders, 2010). In addition, cases failing two or more attention-check items were removed from the dataset for response validity (Meade and Craig, 2012), including “Please select ‘agree’ for this item,” “This is a quality check item. Please choose ‘strongly disagree’ for this item,” and “To ensure you are paying attention, please select ‘neutral.’” After case exclusion, the remaining cases with missing data were handled by full information maximum likelihood (FIML) estimation, providing unbiased parameters assuming that data were missing at random (MAR) to preserve statistical power and reduce bias compared to list wise deletion (Enders, 2010).

Common method bias was examined using Harman’s one-factor test (Podsakoff et al., 2003). Scores derived from Likert-scale items were treated as continuous indicators, consistent with recommended practice when composite scores are used (Kline, 2016). Descriptive statistics (means, standard deviations) and Pearson correlation analyses were conducted using SPSS v27.0 to summarize variable distributions and examine preliminary associations prior to SEM analysis.

The hypothesized mediation model was tested using SEM in Mplus 8.3. The sample size (*N =* 547) was considered adequate for SEM analysis, which exceeded the commonly recommended minimum thresholds for reliable parameter estimation and stable estimation relative to model complexity (Kline, 2016). SEM was selected owing to its ability to simultaneously estimate multiple direct and indirect relationships among latent constructs while accounting for measurement error, making it particularly suitable for testing complex mediation models (Kline, 2016). The model was tested via the maximum likelihood estimator (MLR), which provides robust standard errors and fit indices less sensitive to violations of normality (Kline, 2016). Model fit was evaluated using comparative fit index (CFI), Tucker–Lewis index (TLI), root mean square error of approximation (RMSEA), and standardized root mean square residual (SRMR). No correlated residuals were specified in the model and all measurement errors were assumed to be independent. Model fit was considered acceptable when CFI and TLI values were ≥ 0.90, and RMSEA and SRMR values were ≤ 0.08, consistent with established guidelines (Byrne, 2010; Hair et al., 2017; Hu and Bentler, 1998).

Given that participants were recruited from four different schools, and the primary constructs of this study were at individual rather than the school-level performances, the TYPE = COMPLEX option was used with school as the clustering variable. The standard errors and model fit statistics were therefore adjusted for potential non-independence within the clusters, while the individual-level relationships were still reserved. In addition, participants’ demographic characteristics were included as covariates in the SEM analysis. Gender, parent marital condition, sibling condition, and parents’ education levels were specified as predictors of empathy, prosocial behavior, and academic performance. This approach allows us to examine the hypothesized mediation model while controlling for potential associations of demographics with the socioemotional functioning and academic outcomes.

Indirect effects were tested using bias-corrected bootstrapping with 5,000 resamples and 95% confidence intervals in Mplus 8.3. The indirect effects were considered statistically significant if the confidence intervals did not include zero. Both the specific indirect effects (childhood maltreatment → empathy → academic performance; childhood maltreatment → prosocial behavior → academic performance) and sequential indirect effect (childhood maltreatment → empathy → prosocial behavior → academic performance) were estimated.

## Results

### Common method bias test

The results of Harman’s single-factor test represented that the first factor explained 17.673% of the variance. This was substantially below the commonly suggested 40% threshold, showing that common method bias was not a significant concern in this study (Podsakoff et al., 2003).

### Correlation analysis of variables

The descriptive statistics and Pearson correlation coefficients are displayed in Table 2. Childhood maltreatment was significantly and negatively correlated with empathy (*r* = −0.087, *p* < 0.05), prosocial behavior (*r* = −0.184, *p* < 0.001), and academic performance (*r* = −0.314, *p* < 0.001). Empathy showed a positive correlation with prosocial behavior (*r* = 0.482, *p* < 0.001) and academic performance (*r* = 0.220, *p* < 0.001). Furthermore, prosocial behavior was positively associated with academic performance (*r* = 0.512, *p* < 0.001). These significant correlations provide a basis for the subsequent mediation analysis.

**Table 2:** Descriptive statistics and correlation coefficients of variables (*N =* 547).

	M	SD	1	2	3	4
1. Childhood maltreatment	1.503	0.437	–			
2. Empathy	3.184	0.384	−0.087*	–		
3. Prosocial behavior	3.326	0.714	−0.184**	0.482**	–	
4. Academic performance	2.977	0.542	−0.314**	0.220**	0.512**	–

*p < 0.05, **p < 0.01, ***p < 0.001.

### Structural equation modeling

**Figure 3:**
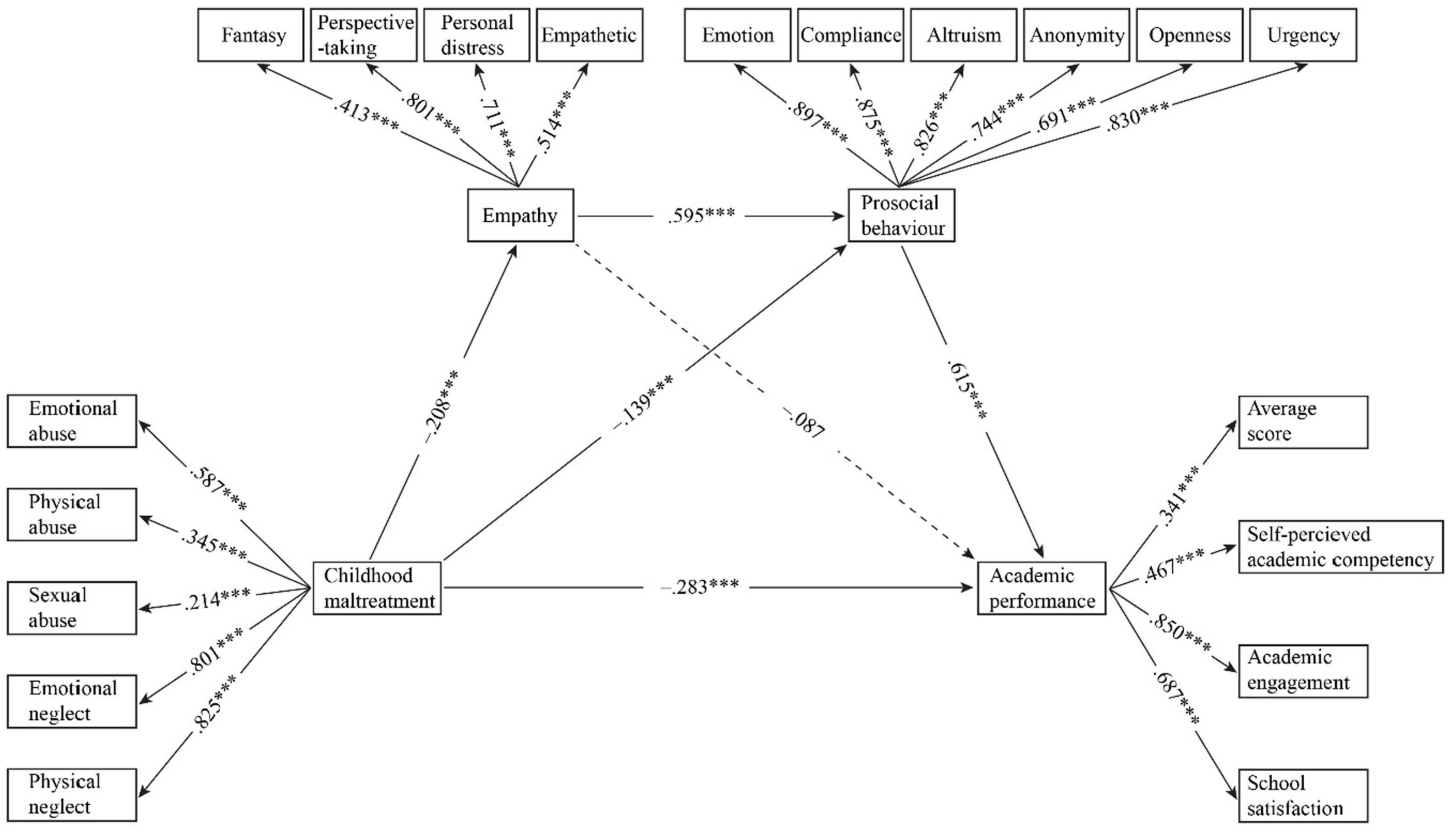
Chain mediation model of variables. *p < 0.05, **p < 0.01, ***p < 0.001.

The hypothesized chain mediation model was tested via SEM (Figure 3). Confirmatory factor analysis (CFA) for the four latent variables and 19 observed variables demonstrated good fit (Table 3).

**Table 3:** Model fit indices for the hypothesized mediation model.

Indices	Model indices values	Standards	Conclusion	Standard sources
*χ*^2^**/**df	4.352	<5 acceptable, <3 excellent fit	Acceptable	<5: Luo et al. (2024)
CFI	0.945	≥0.90	Excellent fit	Byrne (2010)
TLI	0.932	≥0.90	Excellent fit	Byrne (2010)
RMSEA	0.078	≤0.08	Excellent fit	Hair et al. (2017)
SRMR	0.064	≤0.08	Excellent fit	Hu and Bentler (1998)

For direct effects (Figure 3), childhood maltreatment was negatively associated with empathy (*β* = −0.208, *p* < 0.001), prosocial behavior (*β* = −0.139, *p* < 0.001), and academic performance (*β* = −0.283, *p* < 0.001). Empathy was positively associated with prosocial behavior (*β* = 0.595, *p* < 0.001) but was not significantly associated with academic performance (*β* = −0.087, *p* > 0.05). Prosocial behavior positively predicted academic performance (*β* = 0.615, *p* < 0.001). Moreover, the mediation model remained substantively unchanged after controlling for the demographic variables, indicating that the observed relationships were robust to these covariates.

For mediation effects, the bias-corrected bootstrapping with 5,000 resamples revealed significant indirect pathways (Table 4). The total effect of childhood maltreatment was −0.427 (*SE* = 0.053, 95% CI [−0.531, −0.319], *p* < 0.001), with a direct effect of −0.283 (*SE* = 0.052, 95% CI [−0.384, −0.180], *p* < 0.001). The indirect effect through empathy was not significant (*β = 0*.018, *SE* = 0.018, 95% CI [−0.009, 0.063], *p* = 0.303). In contrast, the indirect effect through prosocial behavior was −0.086 (SE = 0.029, 95% CI [−0.143, −0.030], *p* = 0.003), accounting for 20.141% of the total effect. The sequential pathway from empathy to prosocial behavior and then to academic performance demonstrated a significant indirect effect of −0.076 (*SE* = 0.025, 95% CI [−0.133, −0.032], *p* = 0.003), explaining 17.799% of the total effect.

**Table 4:** Direct, indirect, and total effects of the hypothesized model.

Model pathways	Estimated effect (*β*)	Boot SE	Two-tailed *p*-value	95% CI lower	95% CI upper
Direct effect					
Childhood maltreatment – academic performance	−0.283***	0.052	0.000	−0.384	−0.180
Indirect effects					
Childhood maltreatment – empathy – academic performance	0.018	0.018	0.303	−0.009	0.063
Childhood maltreatment – prosocial behavior – academic performance	−0.086**	0.029	0.003	−0.143	−0.030
Childhood maltreatment – empathy – prosocial behavior – academic performance	−0.076**	0.025	0.003	−0.133	−0.032
Total effect	−0.427***	0.053	0.000	−0.531	−0.319

*p < 0.05, **p < 0.01, ***p < 0.001.

Therefore, the effects of childhood maltreatment, prosocial behavior, and the pathway from empathy to prosocial behavior were statistically significant, as indicated by 95% confidence intervals excluding zero. However, empathy alone did not mediate the relationship, with its confidence level including zero.

To conclude, Hypotheses 1, 3, and 4 were supported while Hypothesis 2 was rejected. Childhood maltreatment demonstrated a direct negative association with academic performance. Prosocial behavior independently mediated this association, whereas empathy did not exhibit standalone mediation. Instead, empathy was linked to academic performance through a sequential mediation pathway via prosocial behavior.

## Discussion

### Negative association between childhood maltreatment and academic performance (hypothesis 1)

The negative association between childhood maltreatment and academic performance substantiates the existing international evidence (Slade and Wissow, 2007; Hagborg et al., 2018; Ferrara and Panlilio, 2020). It supports attachment theory’s proposition that disrupted caregiver bonds impair the development of trust and emotional regulation, which are necessary for classroom learning (Bowlby, 1969; Dvir et al., 2014). It also reinforces toxic stress framework by highlighting that compromised executive functioning and memory systems are critical for academic tasks (Shonkoff et al., 2012; Kirke-Smith et al., 2014).

Rural China is characterized by unique educational pressures and resource constraints. The persistence of this association extends the established theoretical frameworks, suggesting the robustness despite its unique socio-educational pressures. The moderate strength of this association aligns with ecological models positioning the intersection of individual, familial, and structural disadvantages in shaping educational trajectories (Slade and Wissow, 2007). However, the substantial unexplained variance requires more comprehensive models incorporating protective factors and additional risk mechanisms, such as how family separation due to labor migration may compound traditional maltreatment effects.

### Nonsignificant mediating effect of empathy (hypothesis 2)

The absence of empathy’s independent mediation role challenges the fundamental assumptions and creates a theoretical paradox: while attachment theory (Bowlby, 1969) positions emotional understanding as foundational to learning capacity, with empirical evidence of Western studies (Dearth-Wesley et al., 2023; Tikkanen et al., 2024), our data suggests that empathy alone carries no academic benefit for maltreated rural Chinese adolescents. This paradox highlights the contextual boundaries and advances theoretical understanding of empathy’s academic relevance.

From a mediation theory perspective (Hayes, 2018), this pattern suggests that empathy may function as a distal antecedent rather than a proximal determinant. While empathy reflects internal emotional understanding, academic outcomes may be more directly shaped by external behaviors. It may benefit academic performance indirectly by facilitating prosocial actions, rather than an independent effect. Empathy appears not as a direct enhancement but a latent capacity requiring behavioral activation. This distinction between internal socioemotional capacity and its behavioral expression provides a theoretical explanation for the nonsignificant independent mediation of empathy alongside a significant sequential mediation pathway. It parallels broader adolescent developmental patterns where internal states diverge from external conducts (Wang and Hawk, 2020).

From a contextual perspective, Western literature emerges from educational environments where emotional intelligence directly translates into academic capital through seminar discussions, group projects, and teacher mentorship (Van Ryzin and Roseth, 2019). These mechanisms presuppose structures with small class sizes enabling interpersonal connection, pedagogical emphasis on collaboration over competition, and assessment systems valuing social–emotional competencies, which are absent in rural Chinese schools. When Qin et al. (2022) find similarly weak empathy-achievement associations among Chinese left-behind children, they attribute it to examination-driven pedagogy that rewards individual performance over peer cooperation. Our finding extends this observation, suggesting that empathy’s academic value depends on institutional recognition and rewards towards emotional competencies. Within rural China’s competitive academic context, adolescents may strategically suppress empathetic responsiveness to maintain focus on individual achievement or channel empathy into instrumental prosocial acts targeting direct academic returns.

### Significant mediating effect of prosocial behavior (hypothesis 3)

The significant mediating role of prosocial behavior extends social learning theory into adverse educational contexts (Bandura et al., 1961). Our finding reveals downstream academic consequences of reduced prosocial behavior, which aligns with recent evidence documenting reduced prosocial tendencies among maltreated youth (Prior et al., 2021) and prosocial behavior’s academic benefits (Carlo et al., 2018; Brass et al., 2024). In under-resourced rural schools where teacher attention remains scarce and instructional quality varies, peer networks provide supplementary academic support through informal tutoring, resource sharing, and collaborative problem-solving (Palacios et al., 2024). Prosocial behavior thus generates academic social capital when formal educational resources fall short.

This significant effect also supports the self-determination theory (Ryan and Deci, 2000) that prosocial engagement satisfies fundamental psychological needs for relatedness, competence and autonomy: helping others reinforces social bonds, consolidating one’s self-perception and behavioral motivation. It further suggests that prosocial behavior associates with not only higher academic achievement but also reduced emotional problems and sustained school engagement (Flynn et al., 2015; Memmott-Elison and Toseeb, 2023). Meanwhile, the relatively modest mediation effect warrants careful interpretation, highlighting prosocial behavior in academic contexts represents as one of the avenues for maltreated adolescents’ need satisfaction and requiring further investigation of the topic.

### Chain mediation via empathy and prosocial behavior (hypothesis 4)

The significant sequential mediation through empathy to prosocial behavior provides crucial theoretical refinement to the empathy-altruism hypothesis (Batson, 1991) in rural Chinese educational context. While empathy failed as an independent mediator, it remains academically relevant when translated into observable social behaviors, reconciling the contradiction between empathy’s theoretical importance and non-significant direct effect. This sequential mediation, contrasted with empathy’s non-significant independent effect, illuminates how competitive educational contexts shape psychological capacities. Empathic capabilities may remain latent unless structured opportunities enable their behavioral expression.

Cross-cultural considerations further enrich this interpretation. While Western research often assumes direct empathy-achievement links through enhanced classroom climate (Dearth-Wesley et al., 2023), our findings suggest that in collectivist and academically competitive contexts, empathy needs to manifest through helping behaviors to yield academic benefits. This cultural specificity does not negate empathy’s importance but rather specifies the conditions under which it becomes academically relevant. The sequential mediation thus represents both a developmental process (internal to external) and a cultural translation (individual feeling to collective behavior).

The similar magnitude of the indirect effects through prosocial behavior alone versus the sequential pathway raises questions about alternative routes to prosocial engagement. Future research should examine whether empathy-driven versus strategically-motivated prosocial behaviors differ in their academic benefits, particularly for maltreated adolescents whose empathic development faces compromise. Examples include strategic reciprocity, compliance with social norms, and instrumental help-seeking.

## Theoretical contributions

This study advances theoretical understanding of maltreatment-achievement relationship. First, our findings extend the theoretical frameworks such as attachment theory, social-learning theory, and self-determination theory beyond their Western origins. While the direct maltreatment-achievement association proves consistency across cultural contexts, the mediating mechanisms reveal important boundary conditions. The non-significant independent role of empathy challenges assumptions embedded in Western theoretical models about direct emotion-achievement links, suggesting that structural and cultural factors fundamentally shape whether and how internal capacities translate into academic outcomes.

Second, the identified sequential pathway refines socioemotional development under adversity. Moving beyond theoretical models treating empathy and prosocial behavior as independent factors, our findings reveal a process whereby maltreatment disrupts empathic development, subsequently constrains prosocial behavior, and ultimately impairs academic performance. It illuminates how socioemotional capacities build upon each other that behavioral manifestations bridge internal states and external outcomes.

Third, our results contribute to ongoing debates on intervention priorities following maltreatment. While the empathy-altruism hypothesis posits emotional understanding as the primary driver of prosocial action, our findings suggest that this sequence is context-dependent. In resource-constrained, achievement-oriented environments, behavioral pathways prove more proximal to academic outcomes than emotional ones.

Finally, the study illuminates the interaction between competitive educational and collectivist cultural values in shaping socioemotional pathways. The persistence of prosocial behavior’s protective function within an individualistic examination system reveals that formal institutional structures and informal social processes operate through intersecting logics. This dual-system perspective extends our understanding how macro-level educational policies and micro-level interpersonal relationships that cooperation could ultimately enhances competition.

## Practical implications

Our findings reveal distinct intervention pathways for addressing maltreatment’s academic consequences for rural Chinese adolescents, with implications for direct prevention and mediating mechanisms. The robust direct association between childhood maltreatment and academic underperformance underscores the primacy of early identification and prevention. Teacher training should emphasize indicator recognition of maltreatment, particularly given that rural adolescents may normalize adverse experiences. Child protection infrastructure is currently underdeveloped but urgently required in these rural regions. Moreover, academic support for maltreated students should extend beyond simple remediation, following the cognitive and emotional load carried by these adolescents.

The non-significant independent role of empathy suggests that emotion-focused interventions alone is insufficient in competitive examination-driven contexts. Interventions should recognize that internal empathic capacity requires behavioral channels to influence academic outcomes. Contrarily, prosocial behavior’s significant mediating role identifies a concrete intervention target. Classroom reconstruction embedding cooperation can be considered, such as group projects, peer tutoring, and collaborative assessments. The sequential mediation highlights the venue of translational interventions bridging emotional understanding and behavioral expression, such as role-playing scenarios where perspective-taking leads to specific helping behaviors, reflection exercises linking emotional awareness to academic mutual aid.

## Limitations and future directions

Several limitations of this study warrant consideration. First, the cross-sectional design restricts causal inference. Although the hypothesized temporal order is grounded in established developmental theories, the present findings reflect associations rather than confirmed causal relationships. Longitudinal and intervention-based studies are needed to more rigorously examine the directionality and to determine whether changes in empathy and prosocial behavior precede improvements in academic performance (Wang and Cheng, 2020). Second, although stratified sampling enhanced representativeness within one province, the findings may not generalize across all rural regions in China. Future studies should replicate the model in other provinces to improve external validity.

Third, the reliance on self-report, particularly retrospective accounts of maltreatment, may introduce potential biases. Although ethical safeguards were implemented, the sensitive nature of maltreatment may still have influenced students’ willingness to disclose experiences. Future research can obtain more comprehensive understanding via multi-informant approaches, such as parents, teachers, or peers (Wienke Totura et al., 2009; Navarro et al., 2020). Finally, the nonsignificant mediation of empathy may reflect measurement limitations or developmental nuances in adolescence. Future research could employ alternative scales or qualitative methods to better capture empathy’s role.

## Data Availability

The raw data supporting the conclusions of this article will be made available by the authors, without undue reservation.
